# Establishment of Three-Dimensional Bioprinted Bladder Cancer-on-a-Chip with a Microfluidic System Using Bacillus Calmette–Guérin

**DOI:** 10.3390/ijms22168887

**Published:** 2021-08-18

**Authors:** Jung Hoon Kim, Seungjin Lee, Su Jeong Kang, Young Wook Choi, Se Young Choi, Joong Yull Park, In Ho Chang

**Affiliations:** 1 Department of Urology, Hanil General Hospital, 308 Uicheon-ro, Dobong-gu, Seoul 01450, Korea; simbauro80@hanmail.net; 2 School of Mechanical Engineering, College of Engineering, Chung-Ang University, 84 Heukseok-ro, Dongjak-gu, Seoul 06974, Korea; leesj09@cau.ac.kr; 3 Department of Urology, Chung-Ang University Hospital, Chung-Ang University College of Medicine, 102 Heukseok-ro, Dongjak-gu, Seoul 06973, Korea; cherubim93@hanmail.net (S.J.K.); ywchoi81@gmail.com (Y.W.C.); urosyc@cau.ac.kr (S.Y.C.); 4 Department of Intelligent Energy and Industry, Graduate School, Chung-Ang University, 84 Heukseok-ro, Dongjak-gu, Seoul 06974, Korea

**Keywords:** urinary bladder neoplasms, BCG vaccine, intravesical administration, 3D bioprinting

## Abstract

Immunotherapy of bladder cancer is known to have favorable effects, although it is difficult to determine which patients will show a good response because of the different tumor microenvironments (TME). Here, we developed a bladder cancer-on-a-chip (BCOC) to mimic the TME using three-dimensional (3D) bioprinting and microfluidic technology. We fabricated a T24 and a 5637-cell line-based BCOC that also incorporated MRC-5, HUVEC, and THP-1 cells. We evaluated the effects of TME and assessed the immunologic reactions in response to different concentrations of Bacillus Calmette–Guérin (BCG) via live/dead assay and THP-1 monocytic migration, and concentrations of growth factors and cytokines. The results show that cell viability was maintained at 15% filling density in circle-shaped cell constructs at 20 μL/min microfluidic flow rate. A 3D co-culture increased the proliferation of BCOCs. We found that the appropriate time to evaluate the viability of BCOC, concentration of cytokines, and migration of monocytes was 6 h, 24 h, and three days after BGC treatment. Lastly, the immunotherapeutic effects of BCOC increased according to BCG dosage. To predict effects of immunotherapeutic agent in bladder cancer, we constructed a 3D bioprinted BCOC model. The BCOC was validated with BCG, which has been proven to be effective in the immunotherapy of bladder cancer.

## 1. Introduction

Reduction of tumor recurrence and progression rate via immunotherapy is one of the most important issues in the treatment of bladder cancer. Bladder cancer is known to be responsive to immunotherapy and topical therapy with Bacillus Calmette–Guérin (BCG), which has been shown to reduce recurrences of high-risk or non-muscle invasive bladder cancer [1]. Metastatic bladder cancer is a suitable candidate for experimenting with more innovative forms of immunotherapy such as new immune checkpoint inhibitors (ICIs). ICIs are monoclonal antibodies that specifically target the inhibitory pathways of the immune system. Interaction between ICIs and their corresponding immune checkpoints increases local and systemic immune responses against tumor cells [2]. Unfortunately, bladder cancer cells are not equally sensitive in their immune response to ICIs because they are related to different individual tumor immune microenvironments.

The tumor microenvironment (TME) refers to the cellular environment in which tumors or cancer stem cells exist [3]; this encompasses the surrounding immune cells, blood vessels, extracellular matrix (ECM), fibroblasts, lymphocytes, bone marrow-derived inflammatory cells, and signaling molecules [4,5]. Interactions between malignant and nonmalignant cells create a TME that affects cancer development and progression [6,7]. The recruitment, activation, and reprogramming of immune and stromal cells in the extracellular space are outcomes of reciprocal interactions between cancer cells and the TME [8,9]. During the initial stages of tumor development, malignant cells in the TME are poor stimulators and poor targets of the immune response [10]. These cells become resistant to the innate immune response and then begin to impair the adaptive immune response [11,12]. However, the composition and structure of the TME, as well as how it shapes cancer development and progression, is believed to vary among patients and types of cancer [13].

BCG is a standard drug for intravesical immunotherapy in patients with bladder cancer. BCG stimulates both innate immunity and inflammatory cancer response to eliminate residual bladder cancer cells [14]. However, BCG immunotherapy alone in patients with non-muscle invasive bladder cancer seems to be insufficient, especially in BCG non-responder groups, and immuno-oncologic drugs are sometimes combined to overcome the low response rate. The immuno-oncologic area in TME and cancer management is focused on ICIs, such as programmed death ligand 1 (PD-L1) on tumor cells and programmed death 1 (PD-1), which are present on normal cells and help maintain the immune response [15]. Immunotherapies based on ICIs tend to shrink tumors; however, the problem with the currently used immunotherapeutic drugs is that their effectiveness is limited and that there are no predictive factors that can forecast their effect [16].

To help predict the response of individual immune-oncologic drugs, we tried to prepare an organ-on-a-chip model. In a previous study, we established a 3D bladder cancer cell culture model using a 3D bioprinter that was more physiologically accurate and stable than the 2D culture model [17]. We successfully designed a single-layered bladder cancer chip using GelMA, which is one of the most versatile hydrogels available for 3D bioprinting technology. It is quite difficult to predict the effect of immune-oncologic agents using traditional 2D single-cell culture. Moreover, it is not an appropriate method to simulate 3D human tissue [18]; therefore, specific 3D co-culture models or animal models are needed. A 3D model has the advantage of being able to represent drug interactions closer to those found in situ and enable the observation of interactions between proteins of the ECM and neighboring cells [19]. In addition, microfluidic devices can improve the survival rate and survival period of cells because they can continuously supply nutrients. Based on the above considerations in bladder cancer therapy, we developed a bladder cancer-on-a-chip (BCOC) that can quantitatively analyze immune response and simulate a tumor immune microenvironment using BCG, an already proven immunotherapeutic drug, via 3D bioprinting and microfluidic technology.

## 2. Results

### 2.1. Performance Validation of BCOC

#### 2.1.1. The Chamber Shape of Bioprinted Cell Construct

In the simulation model, we compared the velocity contours of the circular and square chambers at a flow rate of 20 μL/min. Volume average velocity in the circular and square chambers was predicted to be 17.41 µm/s and 17.49 µm/s, respectively. However, the velocity vector plot on the imaginary cross-section plane (Figure S1) in the chamber shows that the flow stagnates in the corner region and even small vortexes occur. This implies that the cells in the corner region would receive a decreased supplement of the culture medium; it is inferred that, in the circular shape, the nutrients of the culture medium are supplied to the cells of the bioprinted cell block. Therefore, the circular-shaped chamber is a better design for the cells to be cultured than the square-shaped chamber.

#### 2.1.2. The Filling Density and Microfluidic Velocity of BCOC

**Figure 1:**
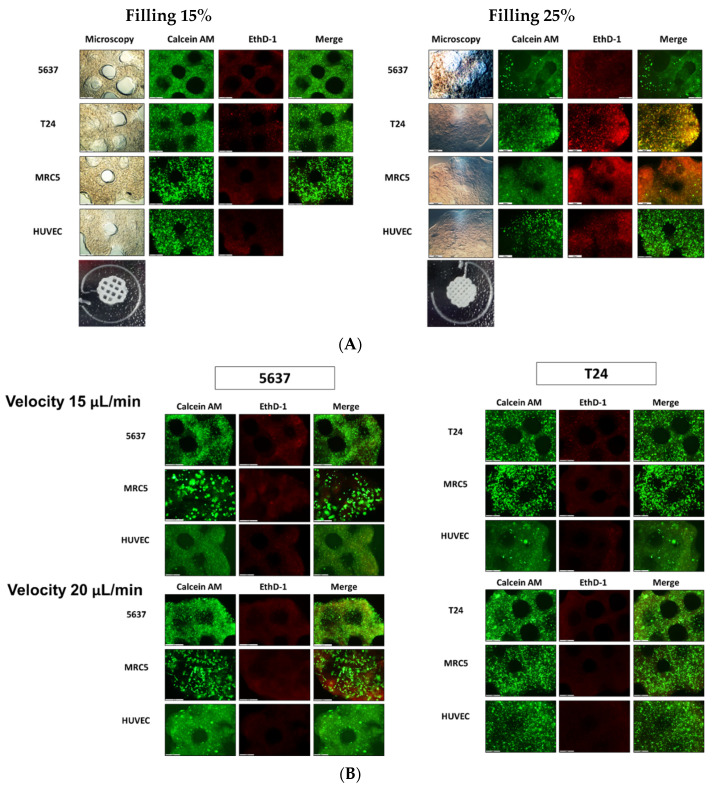

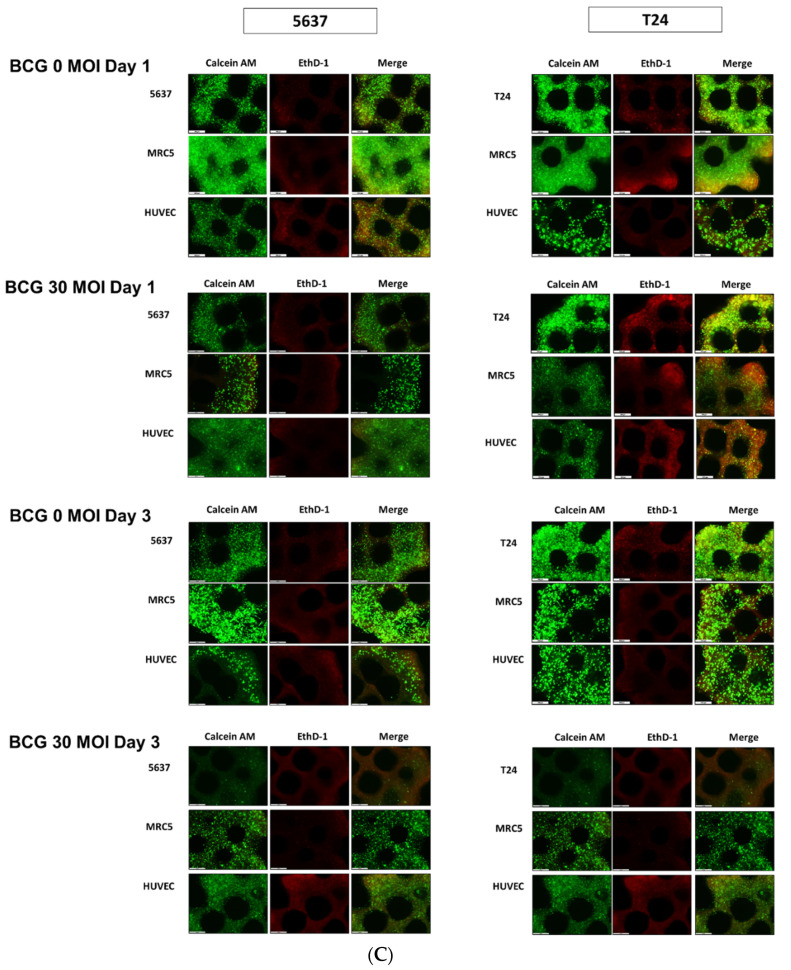
Performance validation of bladder cancer-on-a-chip. The cells were stained with green (live) and red (dead) colors. (Scare bar = 500 μm) (**A**) the GelMA structure and cell viability on BCOC according to structural filling density; (**B**) cell viability on BCOC according to microfluidic velocity; (**C**) bladder cancer cell viability at three days after BCG treatment in BCOC.

We constructed bioprinted cell constructs at 15% and 25% filling densities of cell blocks using 5637, T24, MRC-5, and HUVEC cells. This construct was incubated for three days in growth medium without microfluidic flow. After three days, we found that the holes in 25% filling cell blocks disappeared and melted, although the original patterns were maintained at 15% filling density. Cell viability was also maintained in bioprinted cell constructs under 15% filling densities (Figure 1A). Therefore, we constructed a bioprinted cell with a circular shape and 15% density.

We evaluated cell viability and proliferation rate on 5637 and T24 BCOCs to study the influence of microfluidic velocity at 15 and 20 μL/min. In live and dead staining, the dead cells (red) were prominent in all bioprinted cell constructs in both BCOCs at 15 μL/min velocity (Figure 1B). In the CCK-8 assay, the proliferation rate (mean ± SE, % to 15 μL/min) of T24 (167.5 ± 29.4, *p* < 0.05) and MRC-5 (107.5 ± 5.3, *p* = 0.286) was higher at 20 μL/min velocity (Figure S2). Therefore, we determined the optimal velocity to be 20 μL/min.

#### 2.1.3. Bladder Cancer Cell Viability and Proliferation after BCG Treatment of BCOC

We investigated the cell viability and proliferation rate of 5637 and T24 BCOCs at days 1 and 3 after BCG treatment to determine the optimal culture duration. In live and dead staining, almost all 5637 and T24 cells were dead three days after treatment with BCG at 30 MOI compared with cells that were not treated, although there was no difference after one day (Figure 1C). In the CCK-8 assay, the cell viabilities (mean ± SE, % to day 1 BCG (-)) were decreased after one day in T24 cells (65.3 ± 6.1, *p* < 0.01) and were decreased after three days in both 5637 (21.0 ± 0.43, *p* < 0.01) and T24 cells (14.1 ± 0.4, *p* < 0.01) (Figure S3). Therefore, we decided to evaluate bladder cancer cell viability and proliferation in BCOC three days after BCG treatment.

#### 2.1.4. Concentration of Cytokines after BCG Treatment of BCOC

**Figure 2:**
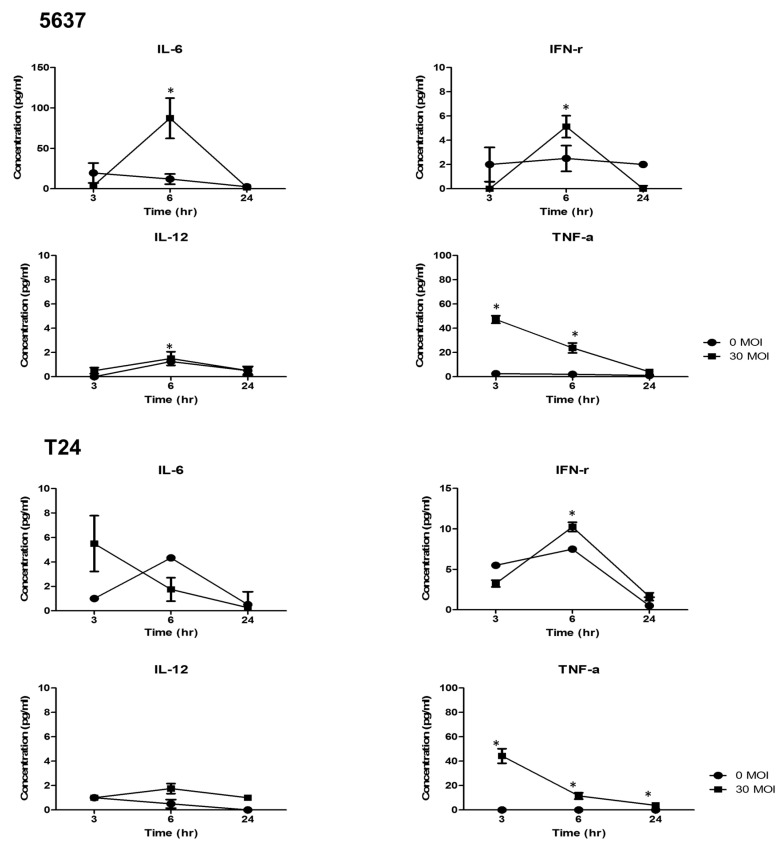
Concentrations of cytokines after BCG treatment of bladder cancer-on-a-chip. Changes of cytokines (IL-6, IFN-γ, IL-12, TNF-α) before and after BCG 30MOI treatment according to time periods in 5637 and T24 cells. We decided that the appropriate measurement time to evaluate immune reaction was 6 h after treatment at bladder cancer-on-a-chip.* indicates *p* < 0.05.

We evaluated the concentration of cytokines (mean ± SE, pg/mL) serially to determine the appropriate time for immune reactions (Figure 2). The concentrations of IL-6 (87.3 ± 25.0, *p* < 0.05), TNF-α (23.8 ± 4.1, *p* < 0.05), IFN-γ (5.1 ± 0.9, *p* < 0.05), and IL-12 (1.5 ± 0.6, *p* < 0.05) at 6 h, and TNF-α (47.3 ± 3.1, *p* < 0.05) at 3 h after treatment of 5637 cells with BCG 30 MOI were higher than those at 3 h after no BCG treatment. After treatment of T24 cells with BCG 30 MOI, the concentration of TNF-α at 3, 6, and 24 h (44.3 ± 6.0, *p* < 0.05; 11.5 ± 2.6, *p* < 0.05; and 3.8 ± 0.5, *p* < 0.05, respectively) and IFN-γ at 6 h (10.3 ± 0.6, *p* < 0.05) were higher than those at 3 h after no BCG treatment. Moreover, the peak time of IFN-γ and IL-12 expression was 6 h and that of TNF-α was 3 h in 5637 and T24 cells; therefore, we decided that the appropriate measurement time to evaluate immune reaction was 6 h after treatment at BCOC.

#### 2.1.5. Chemotaxis of Monocytic THP-1 Cells in Permeable Membrane of BCOC after BCG Treatment

We first evaluated the migration effects of PMA and LPS using a Transwell migration assay. PMA promoted the differentiation and migration of THP-1 cells in a dose-dependent manner; migration of THP-1 cells was potentiated by 20 ng/mL PMA (Figure S4). We also evaluated the migration effects of PMA and BCG on bladder cancer on-a-chip using 5637 cells. The migration rates of THP-1 cells after treatment with BCG at 30 MOI were potentiated by PMA stimulation, although the effects of BCG were minimally increased without PMA stimulation. Therefore, 25 ng/mL was an appropriate concentration of PMA for use in the BCOC (Figure S5).

**Figure 3:**
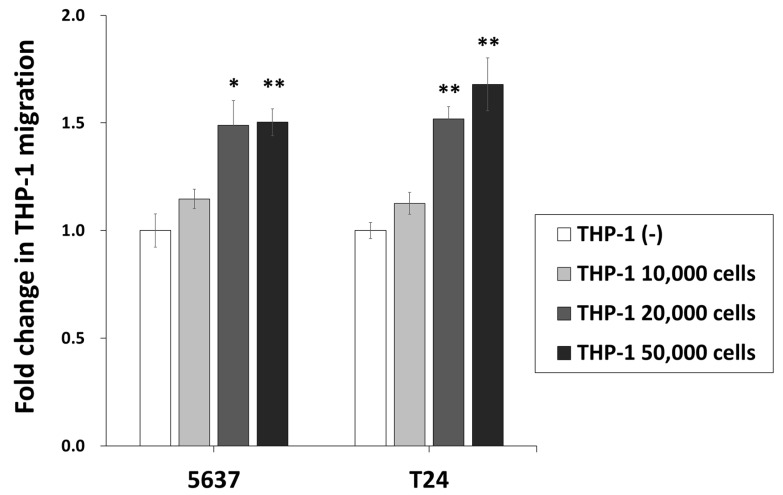
Chemotaxis of monocytic THP-1 cells in permeable membrane of BCOC after BCG treatment according to THP-1 cells number. Data are the mean ± SE of the mean (*n* = 3, per group). * *p* < 0.05, ** *p* < 0.01. SE: standard error.

We evaluated the chemotaxis of monocytic THP-1 cells in the permeable membrane of BCOC after BCG treatment according to the THP-1 cell number (Figure S6). Increasing the number of THP-1 cells in a chip to 2 × 10^4^ and 5 × 10^4^ significantly increased the fold change (mean ± SE, to THP-1 (-)) of 5637 cell migration on a chip to 1.49 ± 0.11 (*p* < 0.01) and 1.50 ± 0.06 (*p* < 0.01), respectively, and of T24 cell migration on a chip to 1.52 ± 0.06 (*p* < 0.01) and 1.68 ± 0.12 (*p* < 0.01), respectively (Figure 3).

### 2.2. The Effect of 3D Microenvironment on Bladder Cancer Cells

**Figure 4:**
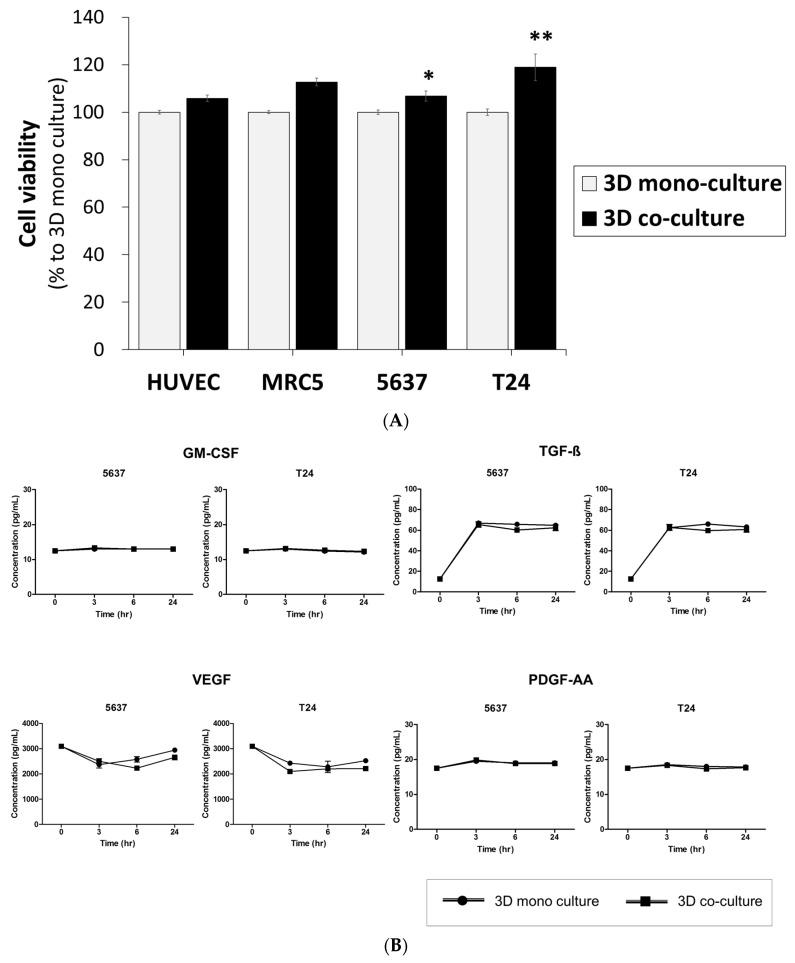
Viability of cells in printed constructs and comparison of 3D mono- and co-culture. (**A**) the viability of the bladder cancer cells in 3D mono- and co-culture; (**B**) growth factors concentrations in 3D mono- and co-culture. Data are the mean ± SE of the mean (*n* = 3, per group). * *p* < 0.05, ** *p* < 0.01. SE: standard error.

We measured the cell viability and proliferation rate of bladder cancer cells to evaluate the effects of 3D co-culture with microfluidics on the BCOC. In the CCK-8 assay, the cell viability (mean ± SE, % in 3D monoculture) of 5637 (106.2 ± 2.2, *p* < 0.05) and T24 (118.9 ± 5.6, *p* < 0.01) cells in 3D co-culture with MRC-5 and HUVEC cells was higher than that in monolayer cultures with microfluidics on a BCOC (Figure 4A). The viability of 5637 and T24 cells in 3D co-culture was similar to that in monocultures with live and dead staining (Figure S7).

We then measured the concentration of growth factors to explore the possible reaction mechanism. However, there was no significant difference between mono- or co-culture with microfluidics for the concentrations of GM-CSF, TGF-ß, VEGF, and PDGF in either 5637 or T24 tumor cells (Figure 4B) (Table S1).

### 2.3. The Evaluation of Immunotherapeutic Effects at BCOC According to BCG Dosage

**Figure 5:**
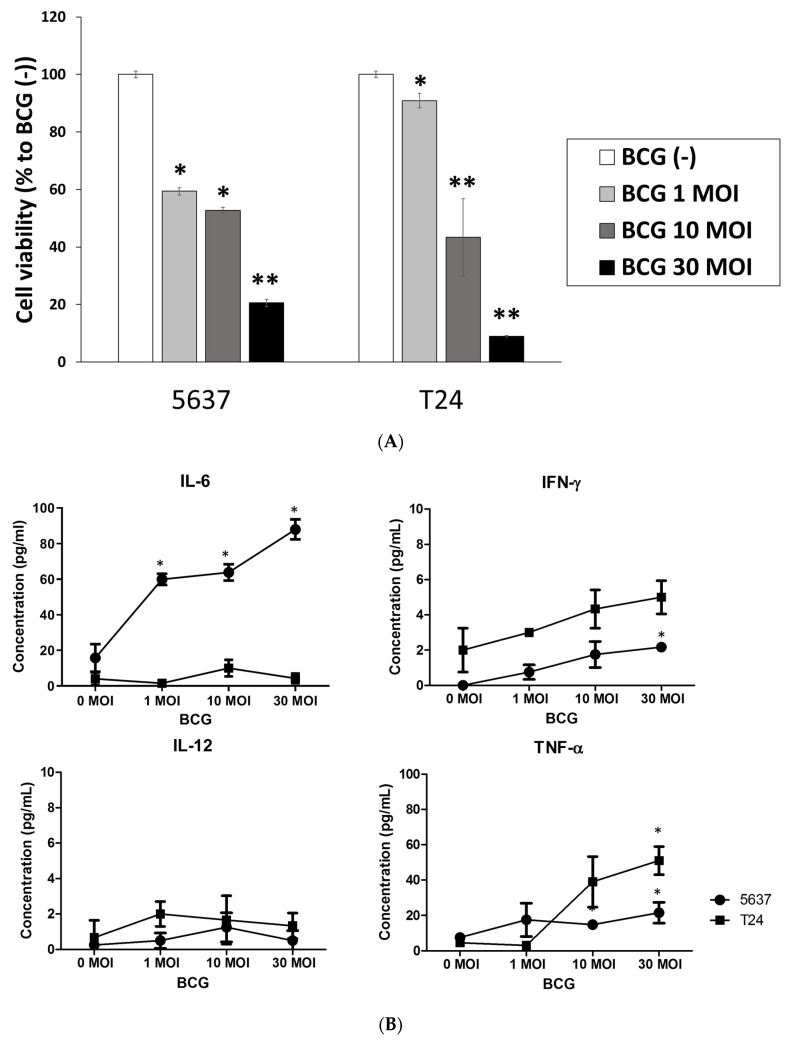

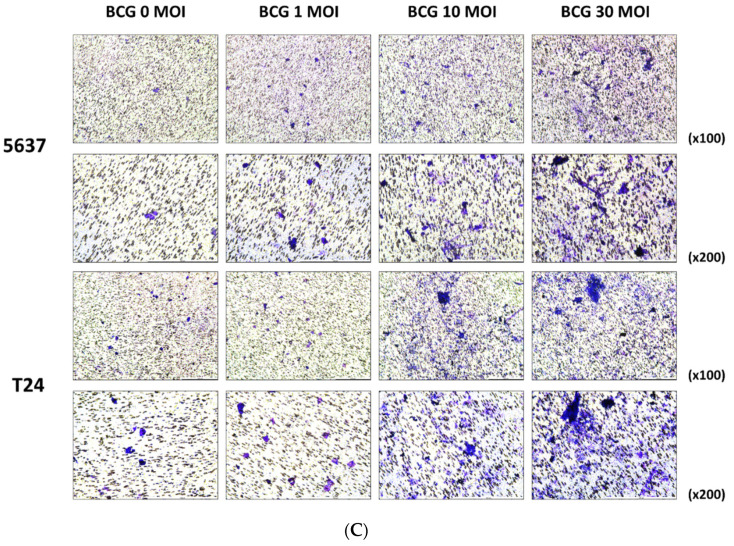
BCG treatment for bladder cancer-on-a-chip. **(A**) Bladder cancer cell viability at three days after BCG treatment at bladder cancer on-a-chip. (**B**) concentrations of cytokines (IL-6, IL-12, INF-γ, and TNF-α) 6 h after BCG treatment at BCOC; (**C**) chemotaxis of monocytic THP-1 cells in permeable membrane 24 h after BCG treatment at BCOC. Data are the mean ± SE of the mean (*n* = 3, per group). * *p* < 0.05, ** *p* < 0.01. SE: standard error.

We evaluated the cell viability and proliferation rate, chemotaxis of monocytic THP-1 cells, and concentration of cytokines simultaneously after treatment with BCG at 1, 10, and 30 MOI to establish the immunologic effects of BCG in BCOC. In the CCK-8 assay, the cell viability (mean ± SE, % to BCG (-)) at three days after BCG treatment was decreased in a dose-dependent manner in the 5637 and T24 BCOCs and significantly decreased in the 5637 BCOC after BCG treatment at 1, 10, and 30 MOI (59.4 ± 1.3, *p* < 0.05; 52.7 ± 1.0, *p* < 0.05; and 20.6 ± 1.3, *p* < 0.01, respectively) and T24 BCOC after BCG treatment at 10 and 30 MOI (43.4 ± 13.5, *p* < 0.05, and 9.0 ± 0.3, *p* < 0.01, respectively) in comparison with that of the control (Figure 5A). In live and dead staining, the viability of 5637 and T24 cells showed a reverse correlation with BCG treatment with increasing MOI (Figure S8).

The concentration of TNF-α increased depending on the dose of BCG used with 5636 and T24 cells. IL-12 expression was increased in the 5637-cell line (Figure 5B). The concentrations of IL-6 (mean ± SE, pg/mL) after treatment with BCG at 1, 10, and 30 MOI (59.9 ± 3.2, *p* < 0.05; 63.9 ± 4.5, *p* < 0.05; and 88.0 ± 5.7, *p* < 0.05, respectively) were higher than those in the control group with 5637 cells. Treatment with BCG at 30 MOI increased the concentration of TNF-α with 5637 (21.5 ± 5.9, *p* < 0.05) and T24 cells (51.0 ± 7.9, *p* < 0.05) and of IFN-γ with 5637 cells (2.2 ± 0.1, *p* < 0.05) (Table S1). The migration rates of THP-1 cells showed increasing patterns according to BCG MOI treatment in 5637 and T24 BCOC (Figure 5C). The fold change in THP-1 cell migration (mean ± SE to BCG (-)) increased dose-dependently according to BCG dosage and significantly increased at BCG 10 and 30 MOI with 5637 cells (1.34 ± 0.04, *p* < 0.05, and 1.48 ± 0.08, *p* < 0.01, respectively) and with T24 cells (1.49 ± 0.03, *p* < 0.05, and 1.56 ± 0.07, *p* < 0.05, respectively) (Figure S9).

## 3. Discussion

In this study, we validated the immunologic effects and mechanisms of the 3D microenvironment for bladder cancer cells and assessed immunotherapeutic reactions according to treatment with BCG. BCOC were fabricated with either T24 or 5637 cells and the MRC-5, HUVEC, and THP-1 cell lines. The results show that cell viability was maintained at a 15% filling density in a circle-shaped chamber and at 20 μL/min microfluidic velocity. The 15% filling density was suitable for maintaining the original shape of the cell block and allowed microfluidics to pass through. There are few studies presenting specific filling densities or microfluidic velocities in organ-on-a-chip model experiments [20]. We investigated UV crosslinking time to determine appropriate exposure time. The cell block was exposed to UV light (356 nm) for 120 s using a UV lamp in order to maintain structural integrity and to survive cultured cells. There may be a concern regarding DNA damage caused by UV exposure. However, it has been reported that DNA double-strand breaks were observed between 2 and 8 h after UV treatment, possibly resulting from replication fork collapse at damaged DNA sites [21]. Therefore, short exposure time of UV will not cause DNA damage. However, the effect of UV light on cytokine expression or THP-1 cell migration has not been analyzed. We have presented specific and intuitive numbers to easily reproduce our results in laboratory conditions. We found that the appropriate time to measure viability and proliferation of bladder cancer cells, secretion of cytokines, and migration of monocytes was three days, 6 h, and 24 h after BCG treatment, respectively. The objective of this experiment was not to investigate the immediate treatment effect of BCG, but to observe a delayed immune response. BCG is known to induce a robust innate immune response locally and systemically [22]. Following initial BCG instillation, cytokine and chemokine concentrations have been shown to peak within 2–8 h, leading to immune cell recruitment to the urothelium. This innate response is further characterized by granuloma formation in the bladder wall, containing macrophages, dendritic cells, lymphocytes, neutrophiles, and fibroblasts [23,24]. These published results were consistent with our experimental data. Therefore, we suggested that it is appropriate to measure the viability and proliferation of bladder cancer cells at three days, concentration of cytokines at 6 h, and migration of monocytes at 24 h.

Human THP-1 monocytes are differentiated into macrophages by incubation in the presence of PMA [25]. Based on the literature, we experimented with different concentrations of PMA and LPS and observed that PMA was superior to LPS in differentiating monocytes into macrophages.

We found that the proliferation of bladder cancer cells was increased under the 3D microenvironment; however, the concentrations of growth factors did not significantly vary. The growth medium used in the experiment was endothelial cell basal medium-2 (EBM-2), which contains 12 kinds of growth factors. Therefore, it is possible that changes in growth factor concentration were mixed with those in the culture medium and masked, so that there was no noticeable difference. Additional experiments with culture medium where targeted growth factors have been removed or growth factor-free culture medium may be required in the future.

When comparing mono- and co-cultures, the experiments related to growth factors showed several differences from our expectations. We expected that the secretion of growth factors would increase when bladder cancer cell lines were cultured with HUVEC and MRC-5 cells compared with when bladder cancer cell lines were cultured alone. However, similar levels of growth factors were detected in both the mono-and co-cultures. This could be interpreted to mean that the growth factors secreted in the two groups were too low to measure. Although the growth factors were measured correctly, they may not be clearly distinguished because of the influence of EBM-2 media. We believe that the high VEGF concentration value from the initial measurement was also influenced by the culture medium.

In the experiment where the dose of BCG was changed, we showed that, as the concentration of BCG increased, the proliferation of bladder cancer cells was inhibited in a dose-dependent manner. This means that the condition of the BCOC has been successfully established by examining the dose-dependent response of BCG, which is already recognized as the standard of immunotherapeutic drug treatment.

Bladder cancer is heterogeneous in terms of immunotherapeutic sensitivity, which leads to uncertainty in the treatment effects of individual therapies. Testing ICI drug sensitivity in individual patients is critical and significant for personalized therapies. Response to ICIs depends on multiple factors, including the molecular characteristics of the tumor and the interaction with the immune system [26]. Both BCG instillation and ICI administration are effective immunotherapies for bladder cancer. This specific feature of bladder cancer enables simple local and systematic immunotherapy applications. Combination treatments are being explored for different stages of bladder cancer. Phase 2 CheckMate 9UT is currently enrolling patients with BCG-unresponsive non-muscle invasive bladder cancer, who will be treated with nivolumab ± the IDO-1 inhibitor BMS986205 ± intravesical BCG [27]. We believe that our BCOC can help in screening the combined effects of BCG and ICI.

**Figure 6:**
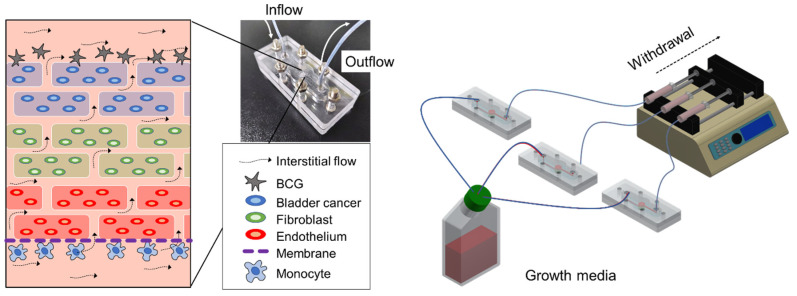
Schematic representation of three-dimensional bioprinted bladder cancer-on-a-chip with microfluidic system using Bacillus Calmette–Guérin.

Bioprinting technology has several advantages over other 3D based culture. Bioprinting can produce various cancer models by changing the type of cells to be injected; the shape can be made as desired, and mass production is also possible. In particular, the BCOC model can easily evaluate drug reactions as this can perform three different tests simultaneously, as shown in Figure 6. Differences in drug effects between 2D and 3D cultures have been reported in bladder cancer models. Kim et al. showed that the effect of BCG and rapamycin was more exaggerated in 2D cell cultures than in 3D cell culture environments [17]. Culture of RT4 cells, a bladder cancer cell line, under 3D conditions also showed higher resistance to doxorubicin compared with that in 2D cultures [28]. The microfluidic environment is known to have a positive effect on the expression of in vivo characteristics of cells. Most cells in the body are exposed to interstitial fluid, and biomolecules secreted from the cells are transferred to the surroundings by diffusion rather than by the convection of body fluids [29]. Interstitial fluid flow is necessary for correct cellular proliferation and growth, and physiological, pathological, and developmental processes as well as blood flow. In these studies, the 3D culture model reflects the microenvironment of the human body better than the 2D culture model. Thus, our device can be used to study the effects of the interstitial flow and gradient levels. Therefore, the microfluidic chip is an efficient experimental platform that united numerous experiments into one chip and realize 3D co-culture [30].

This study has several limitations. First, we have not been able to provide an appropriate tool to assess the maturation of 3D microenvironments. Before measuring the level of several growth factors to evaluate maturation, growth media that does not contain specific growth factors should be used. Second, the current analysis methods have the disadvantage of destroying the BCOC. If we develop a method to study the chip without causing damage, we can enable continuity of the experiment. To avoid damage to the continuity, various non-invasive methods such as Raman or impedance spectroscopy can be considered. Third, this model did not clearly separate blood and urine, unlike the actual urinary bladder. In the bladder, the urinary space and blood vessels are separated by the bladder wall. To overcome this issue, we will create an advanced organ-on-a-chip system that contains a physical barrier. Fourth, this study was based on some cell lines that are frequently used in cell level experiments. There are many challenges to applying single patient-derived cancer cells, fibroblasts, and endothelial cells. Since we are still at the early stage, we focused on the effectiveness of BCOC by reinforcing the experimental conditions. In the future, we intend to use patient-derived autologous lymphocytes, fibroblasts, stromal cells, and cancer cells. Finally, lymphocytes were not treated in the chip to assess the immune reactions to ICIs. Since lymphocytes were not treated in the chip, the immune effect of ICIs could not be accurately measured under the experimental conditions. We plan to use ICIs, not BCG, to treat lymphocytes in the next experiment.

## 4. Materials and Methods

### 4.1. Cells and Reagents

T24 and 5637 cells (human bladder cancer cell line), MRC-5 cells (human lung fibroblast cell line), and THP-1 cells (human leukemia monocytic cell line) were purchased from the Korean Cell Line Bank (KCLB, Seoul, Korea). THP-1 cells are widely used for immune cell migration assays employing cell lines. THP-1 is a human monocytic cell line derived from an acute monocytic leukemia patient. It is used to differentiate into macrophage-like cells. Human umbilical vein endothelial cells (HUVECs) were purchased from Lonza (Basel, Switzerland). T24 cells were cultured in RPMI-1640 supplemented with 10% fetal bovine serum (FBS) and 1× penicillin/streptomycin (Gibco, Gaithersburg, MD, USA). MRC-5 cells were cultured in MEM supplemented with 10% FBS and 1× penicillin/streptomycin (Gibco, Gaithersburg, MD, USA). THP-1 cells were cultured in RPMI-1640 supplemented with 10% FBS, 1× penicillin/streptomycin, and 0.05 mM 2-mercaptoethanol (Gibco, Gaithersburg, MD, USA). HUVECs were cultured in EBM-2 Bulletkit (Lonza, Basel, Switzerland). We compared several culture media, such as EBM-2, MEM, and RPMI 1640, to select a proper culture media for BCOC. We found that 5637, T24, and MRC-5 cells are more viable in EBM-2 media (Figure S10). All cultures were maintained in a humidified atmosphere at 37 °C and 5% CO_2_. BCG was obtained as a commercial lyophilized preparation (OncoTICE, Merck Sharp and Dohme, Kenilworth, NJ, USA). BCG was resuspended in phosphate-buffered saline (PBS; Hyclone, Logan, UT, USA), and aliquots with a multiplicity of infection (MOI) of 100 (1 × 10^8^ cells/mL) were prepared and stored at –80 °C until use. Gel4Cell (Innoregen, Daegu, Korea) was used as the GelMA prepolymer solution.

### 4.2. Fabrication of BCOC with a Microfluidic System

**Figure 7:**
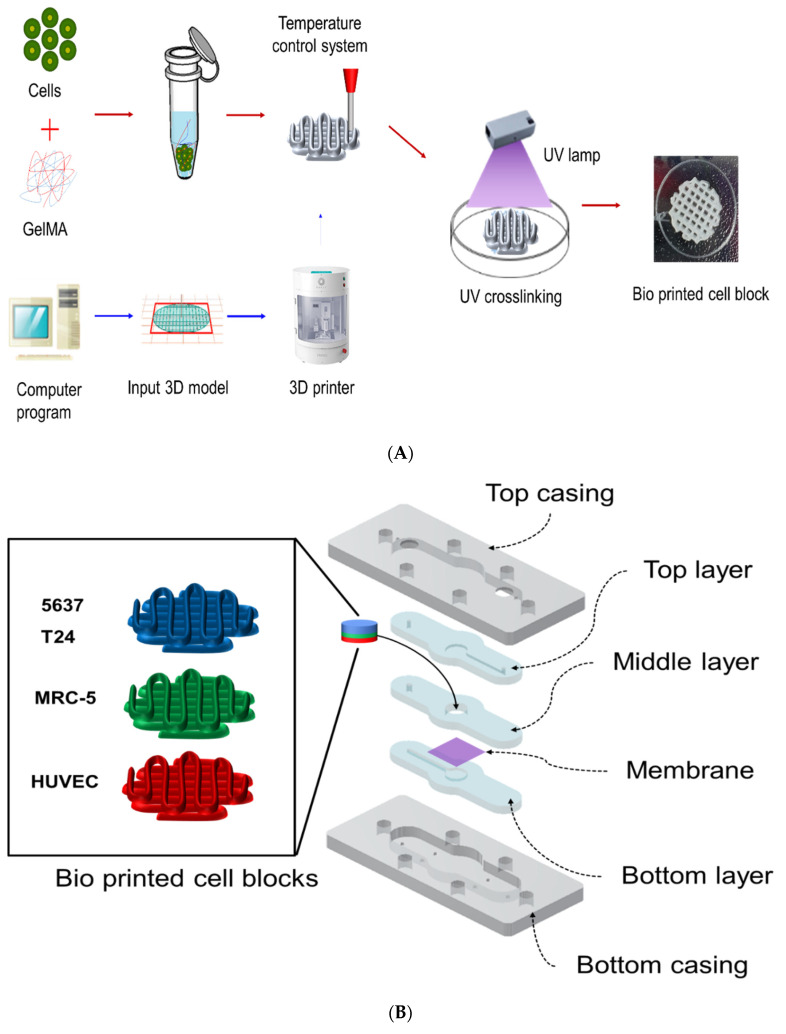

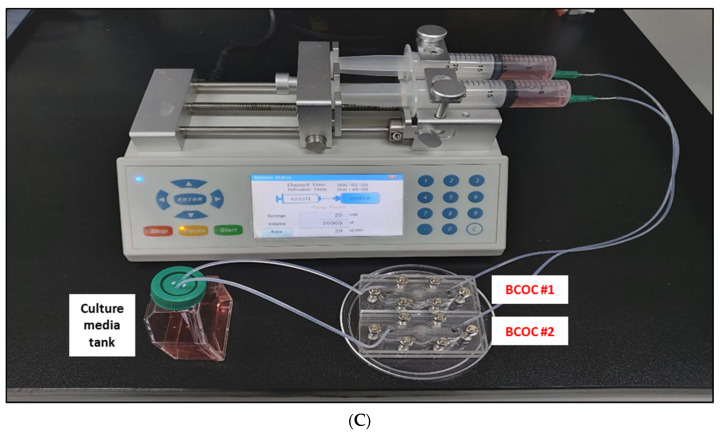
The concept of bladder cancer-on-a-chip. (**A**) The process to make the bioprinted cell block using a 3D bioprinter; (**B**) schematic illustration of the whole structure of the device; (**C**) the microfluidic device connected to the perfusion equipment.

The 3D cell construct was fabricated using an EDISON Invivo^®^ 3D bioprinter (ROKIT Healthcare, Seoul, Korea). In addition, 5637, T24, MRC-5, and HUVEC cells at a density of 5 × 10^5^ cells/mL were collected by centrifugation at 1300 rpm for 3 min and suspended in Gel4 Cells. The mixtures were placed into a syringe with a 27-gauge Teflon needle and then loaded into the 3D bioprinter (nozzle size: 0.2 mm; printing speed: 5 mm/s; X, Y = 6 mm, Z = 1 mm; chip Z = 3 mm; filling density: 15 or 20%). After 3D bioprinting, the 3D cell construct was physically crosslinked by exposure to UV light (356 nm) for 120 s. After further crosslinking, the 3D cell construct was incubated with culture medium for 48 h (Figure 7A).

In the 3D BCOC, a complex 3D cell structure was prepared consisting of bladder cancer cells (5637 or T24), stromal fibroblasts (MRC-5), and endothelial cells (HUVEC) (Figure 7B). The top casing and bottom casing were fabricated by engraving acrylic plates using a milling machine (DAVID 3040; David Motion Technology, Incheon, Korea). To fabricate the microfluidic channel parts (top, middle, and bottom layers) with polydimethylsiloxane (PDMS), the acrylic molds were sculpted using the milling machine. Then, the uncured PDMS mixture of a prepolymer and a curing agent in a 10:1 ratio (Sylgard^®^ 184; Dow Inc., Midland, MI, USA) was filled into the prepared acrylic mods and cured for 2 h at 80 °C on a hot plate. Physiological microarchitecture was recapitulated in the BCOC microdevice with two three cell-culture chambers separated by a polycarbonate track-etched (PCTE) membrane (GVS filter technology, Sanford, ME, USA). BCOC was treated with BCG on the top layer and monocytes at the bottom layer for 2 h without microfluidics. The microfluidic device consisted of a tissue culture platform, nutrient supply channel, and waste removal chamber (Figure 7C). The bottle on the lower left of the figure is a culture media tank and the culture media flows inside the BCOC at a constant flow rate using a syringe and a mechanical device. The microfluidic channel comprised a micro channel in the bottom layer, a micro channel in the top layer, and a chamber in the middle layer for the bioprinted cell block. The height and width of each micro channel are both 1 mm, and the diameter and height of the chamber are 6 mm and 3 mm, respectively (Figure S11).

### 4.3. Live/Dead Staining Assay

The cell survival rate in the 3D cell constructs was assessed on days 1 and 3 after bio-fabrication and BCG treatment. A fluorescent live/dead staining solution (Thermo Fisher Scientific, Waltham, MA, USA) was used according to the manufacturer’s instructions. Each 3D-cell construct and 2D-cultured cells were washed in Dulbecco’s phosphate buffered saline (DPBS) three times before staining. The DPBS mixture with Calcein-AM (2 µM) and EthD-1 (4 µM) was filtered through a 0.22-mm syringe filter (Sigma, St. Louis, MO, USA). Cell morphologies were observed under a fluorescence microscope (DMI8; Leica, Wetzlar, Germany). Three independent samples were analyzed.

### 4.4. Cell Proliferation Assay

A Cell Counting Kit-8 (CCK-8; Dojindo, Kumamoto, Japan) was used to analyze cell proliferation in 3D-cell constructs on days 1 and 3, according to the manufacturer’s instructions. The 3D cell constructs were washed three times with DPBS. Then, 1 mL of DPBS and 0.1 mL CCK-8 solution were added to each 60-mm cell culture dish and incubated in the dark for 8 h with 5% CO2 at 37 °C. After incubation, 0.2 mL was transferred to a 96-well plate. The absorbance of each well was measured at 450 nm using a microplate reader (SpectraMax i3x; Molecular Devices, Sunnyvale, CA, USA). Three independent samples were tested in each group.

### 4.5. Migration Assay

Phorbol 12-myristate 13-acetate (PMA; Sigma, St. Louis, MO, USA) or lipopolysaccharide (LPS) were used to differentiate THP-1 cells to amplify the immune reaction; the migration effects of PMA and LPS were evaluated using a Transwell migration assay. Briefly, the basement membranes of Boyden chambers were rehydrated with 300 μL serum-free RPMI, and 2 × 10^4^ THP-1 cells and PMA or LPS were then seeded into the upper area of the chamber in serum-free RPMI medium. The bottom wells were filled with 5 × 10^5^ 5637 cells and RPMI supplemented with 10% FBS. After 24 h of incubation (37 °C, 5% CO_2_), non-migratory cells were removed from the upper chamber, and the cell migration was assessed by light microscopy after staining the migrated cells with a Crystal Violet Cell Stain Solution (Cell Biolabs, San Diego, CA, USA).

THP-1 monocytes were differentiated into macrophages by incubation for 24 h in RPMI 1640 medium with PMA. Differentiated THP-1 cells were seeded in the bottom layer of the BCOC. After that, the PCTE membrane (GVS filter technology, Sanford, ME, USA) was mounted, 3D cell constructs were stacked on the second layer, and then BCG was added. After stabilizing for 2 h for the immune response, EBM-2 medium (Lonza Basel, Switzerland) was pumped into the chip. After 24 h, the PCTE membrane was fixed with 4% paraformaldehyde (Sigma, St. Louis, MO, USA), followed by chemical staining with 0.1% crystal violet. Positive THP-1 staining was visualized using an Olympus CKX41 inverted microscope (×100 and ×200; Olympus, Tokyo, Japan). Three equal-sized fields were randomly selected for THP-1 cell counting, and the average was calculated. Data are presented as the mean ± SE of the mean (*n* = 3 per group). * *p* < 0.05 vs. Control.

### 4.6. Measurement of Cytokines and Growth Factors

The growth media after 3, 6, and 24 h of biofabrication and BCG treatment was collected in a syringe for each fluidic culture solution of BCOC and centrifuged at 3000 rpm for 10 min at 4 °C. The supernatant was aliquoted and stored at −80 °C until analysis. The levels of cytokines in the BCOC cultured supernatant were analyzed using a Luminex assay (R&D Systems, Minneapolis, MN, USA) and MAGPIX^®^ system (Luminex, Austin, TX, USA) according to the manufacturer’s instructions. The growth factors analyzed were tumor growth factor (TGF)-β1 (LTGM100), granulocyte-macrophage colony-stimulating factor (GM-CSF) (LUXLM215), platelet-derived growth factor (PDGF)-AA (LUXLM221), and vascular endothelial growth factor (VEGF) (LUXLM293). The analyzed cytokines were tumor necrosis factor (TNF)-α (LUXLM210), interleukin (IL)-6 (LUXLM206), IL-12 (LUXLM219), and interferon (IFN)-γ (LUXLM285). The supernatant was diluted 2× prior to assaying for TGF-β1, GM-CSF, PDGF-AA, VEGF, TNF-α, IL-6, IL-12, and IFN-γ. Standard curves were generated using serial dilutions of assay standards for quantification. The Bio-Plex Manager 6.1 software for MAGPIX was used for bead acquisition and analysis of median fluorescence intensity (MFI). The concentrations of the released cytokines and growth factors in each tube were calculated by subtracting the values of the basal media.

### 4.7. Simulation Model

To investigate the flow environment that cells in our system were exposed to, a numerical analysis was performed using ANSYS Fluent 19.2 (ANSYS, Inc., Canonsburg, PA, USA), a commercial computational fluid dynamics (CFD) tool. Two different geometries of the bioprinted cell blocks were tested, and circle and square shapes were compared. The diameter of the circle shape was 6 mm, and the square shape was 6 mm long on one side. The bioprinted cell blocks and channel parts were 3 and 1 mm thick, respectively (Figure S12A). As a working fluid, the properties of water at 37 °C temperature (density set to 993.3 kg/m^3^) were assumed for the culture media, and the outlet was set to 20 μL/min mass flow outlet condition. The porous structure of the bioprinting cell block was established using a porous medium with a constant porosity of ε = 0.99 and a viscous resistance of R = 1.33 × 10^13^/m^2^. Approximately 523,284 hexahedral meshes constitute the computational domain (Figure S12B). The channel was assumed to have a steady-state flow, and each case was calculated with 10,000 iterations; the residuals of all cases were under 10^−6^.

### 4.8. Statistical Analysis

All data are presented as the mean ± standard error of the mean (SEM) of at least three individual experiments performed in triplicate. Data were compared using Student’s *t*-test. Statistical significance was set at *p* < 0.05.

## 5. Conclusions

We constructed a 3D bioprinted BCOC model with a microfluidic channel to predict immunotherapeutic effects. BCOC has been validated through BCG treatment, which has proven to be effective in the immunotherapy of bladder cancer. The final goal of this system is to apply patient-derived cancer cells for personalized medicine. This will promote the development of precision medicine for tumor immunotherapy. There is considerable expectation in the field of research on the effects of combination therapy with ICIs and chemotherapeutic agents. If combination therapy is studied using patient-oriented cancer cells, it will be an effective tool for analyzing drug response.

## Data Availability

Not applicable.

## Supplementary Materials

The following are available online at https://www.mdpi.com/article/10.3390/ijms22168887/s1, Figure S1: Comparison of the velocity vectors in the circular shaped and square shape chambers, Figure S2: GelMA structure and GelMA/cells 3D culture models according to microfluidic velocity, Figure S3: Bladder cancer cell viability on days 1 and 3 after BCG treatment at BCOC, Figure S4: Migration of differentiated THP-1 cells (2 × 10^4^ cells) by phorbol 12-myristate 13-acetate (PMA) or lipopolysaccharide (LPS) at Transwell migration assay after 24 h, Figure S5: The migration of differentiated THP-1 cells by PMA after BCG treatment in BCOC, Figure S6: Chemotaxis of monocytic THP-1 cells in permeable membrane of bladder cancer-on-a-chip after BCG treatment according to THP-1 cell number, Figure S7: GelMA structure and viability of the bladder cancer cells in 3D mono- and co-culture, Table S1: Concentrations of growth factors in 3D mono- and co-culture, Figure S8: Bladder cancer cell viability measured by live/dead staining at 3 days after BCG treatment at BCOC, Figure S9: Migration rate of monocytic THP-1 cells in permeable membrane 24 h after BCG treatment at BCOC, Figure S10: Comparison images of EBM-2 and other culture media for each cell lines, Figure S11: Microfluidic channel of BCOC, Figure S12: Computational domain of the BCOC microfluidic channel.
