# Supplementary material for: Establishment of Three-Dimensional Bioprinted Bladder Cancer-on-a-Chip with a Microfluidic System Using Bacillus Calmette–Guérin
Source: Int J Mol Sci. 2021 Aug 18;22(16):8887. doi: 10.3390/ijms22168887 (PMC8396314; doi:10.3390/ijms22168887)

Supplementary Materials

Establishment of Three-Dimensional Bioprinted Bladder Cancer-on-a-Chip with a Microfluidic System Using Bacillus Calmette–Guérin

Figure S1. Comparison of the velocity vectors in the circular- and square-shaped chambers. Cross section plain is located horizontally in the middle of the bio printed cell block. The flow is formed more uniformly in a circular chamber than in a square chamber. In a circular-shaped chamber, the medium passes without a vortex, whereas in a square-shaped chamber, a vortex occurs in the corner region.

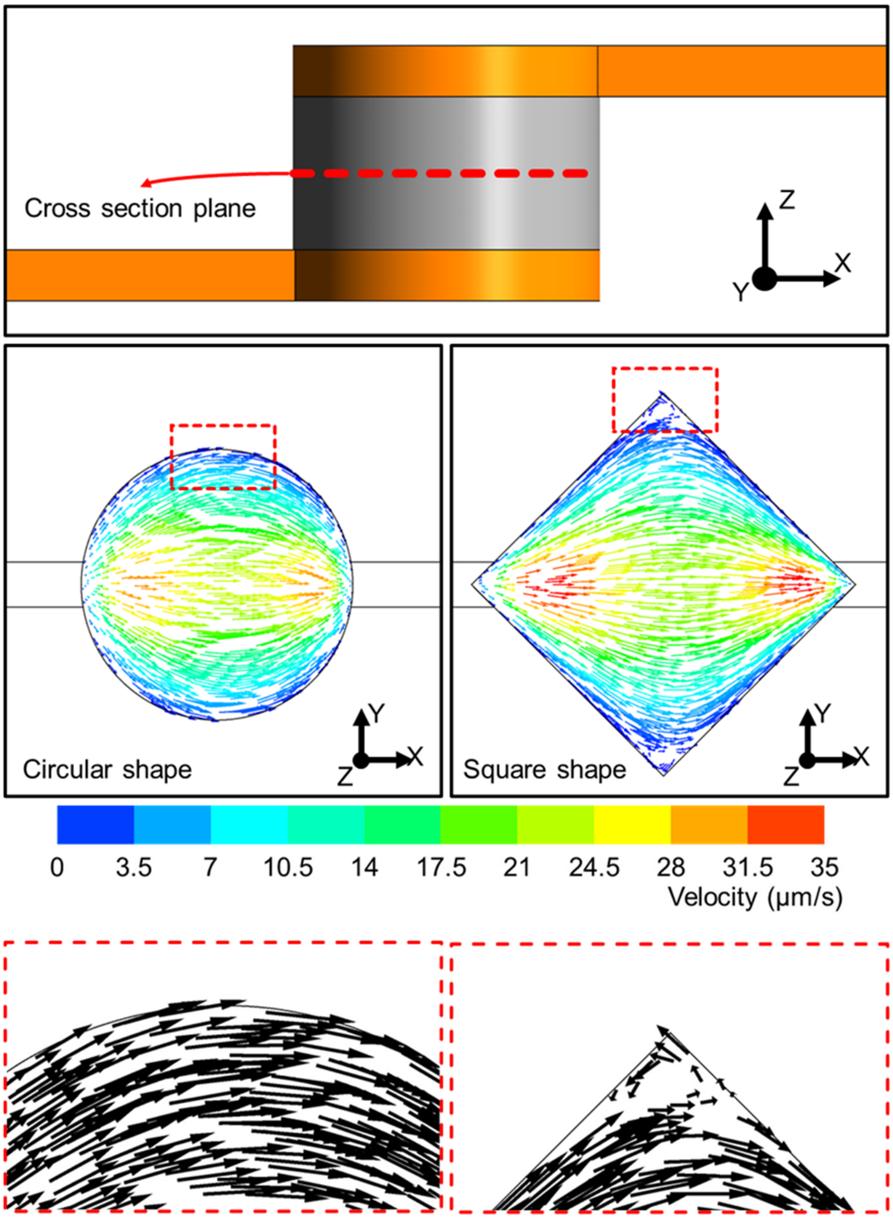

Figure S2. GelMA structure and GelMA/cells 3D culture models according to microfluidic velocity.

Data are the mean ± SE of the mean (n = 3, per group). *$p < 0.05$. SE: standard error.

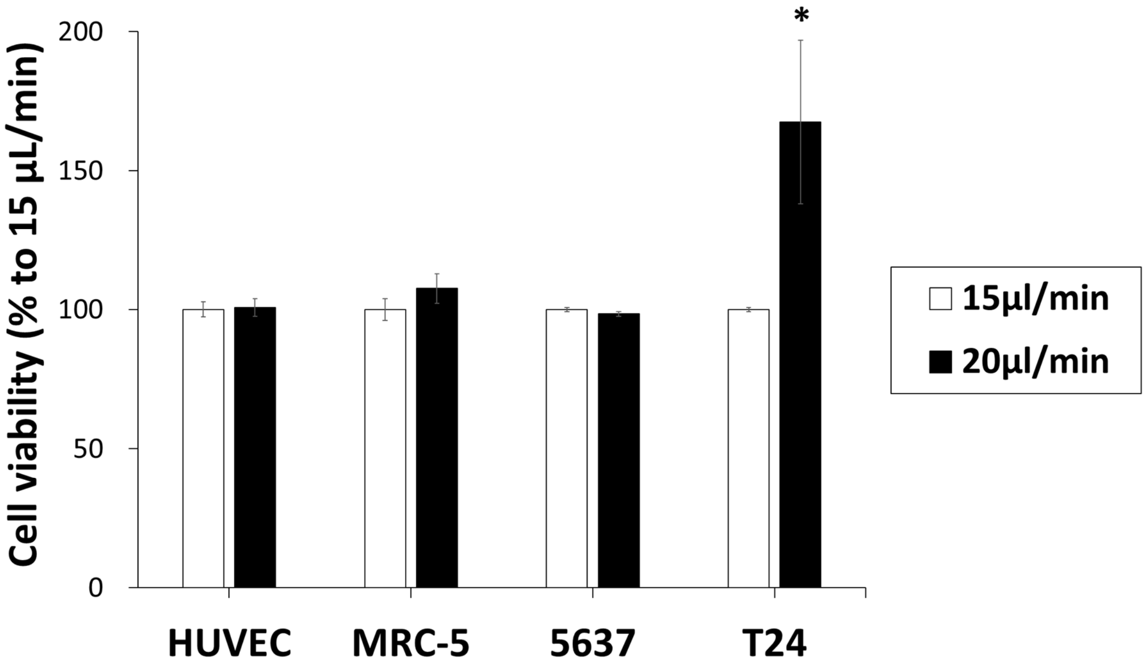

Figure S3. Bladder cancer cell viability on days 1 and 3 after BCG treatment at BCOC. Data are the mean ± SE of the mean (n = 3, per group). *$p < 0.05$, **$p < 0.01$. SE: standard error.

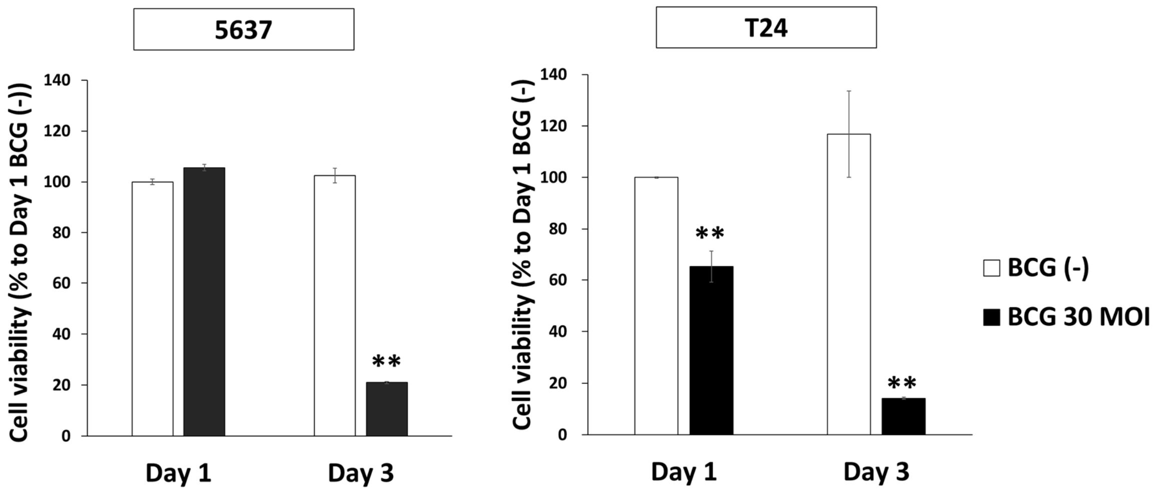

Figure S4. Migration of differentiated THP-1 cells (2 × 10^4 cells) by phorbol 12-myristate 13-acetate (PMA) or lipopolysaccharide (LPS) at Transwell migration assay after 24 h.

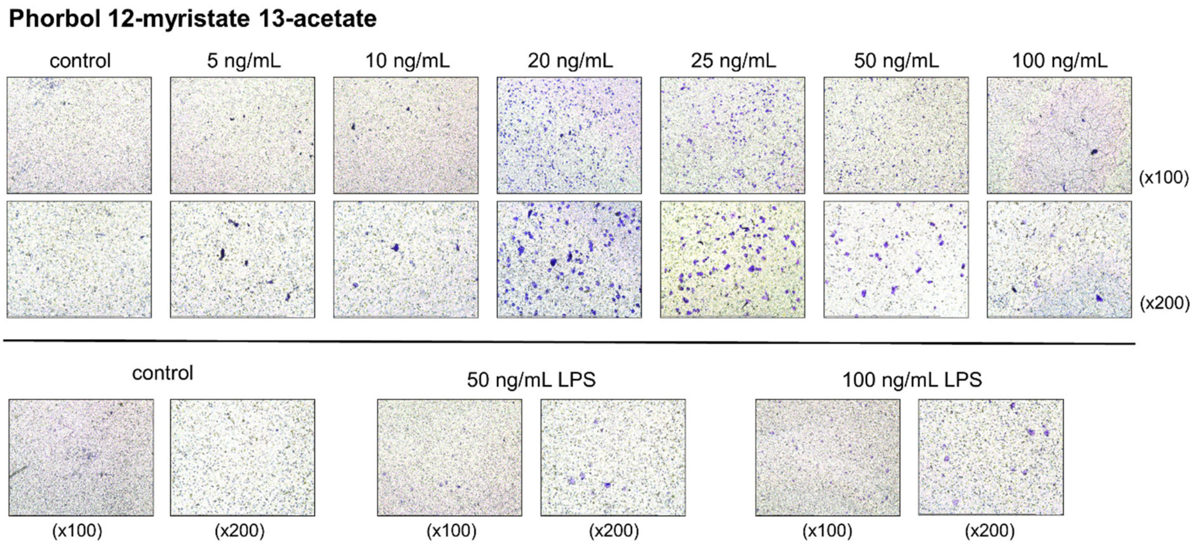

Figure S5. The migration of differentiated THP-1 cells by PMA after BCG treatment in BCOC.

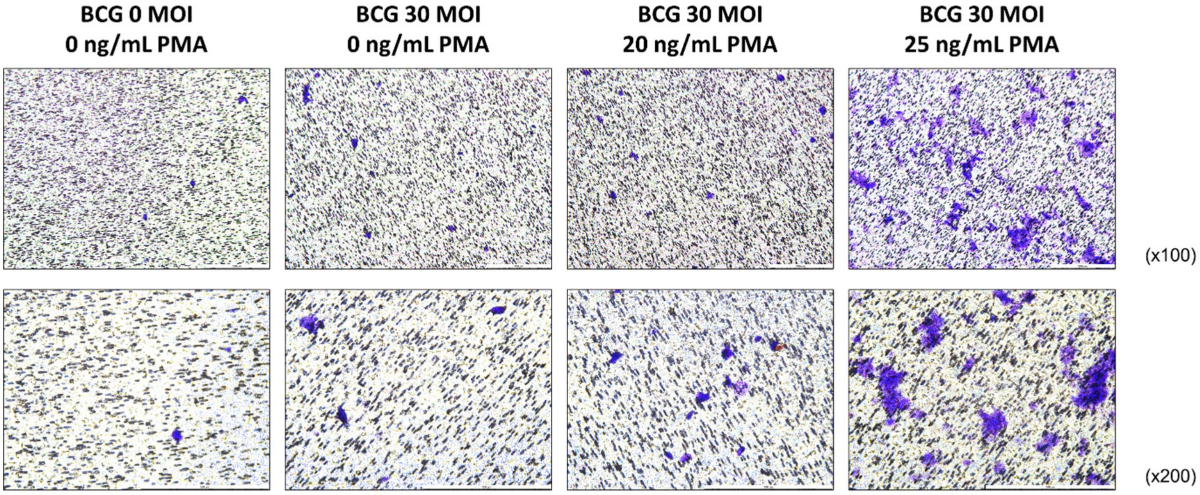

Figure S6. Chemotaxis of monocytic THP-1 cells in permeable membrane of bladder cancer-on-a-chip after BCG treatment according to THP-1 cell number.

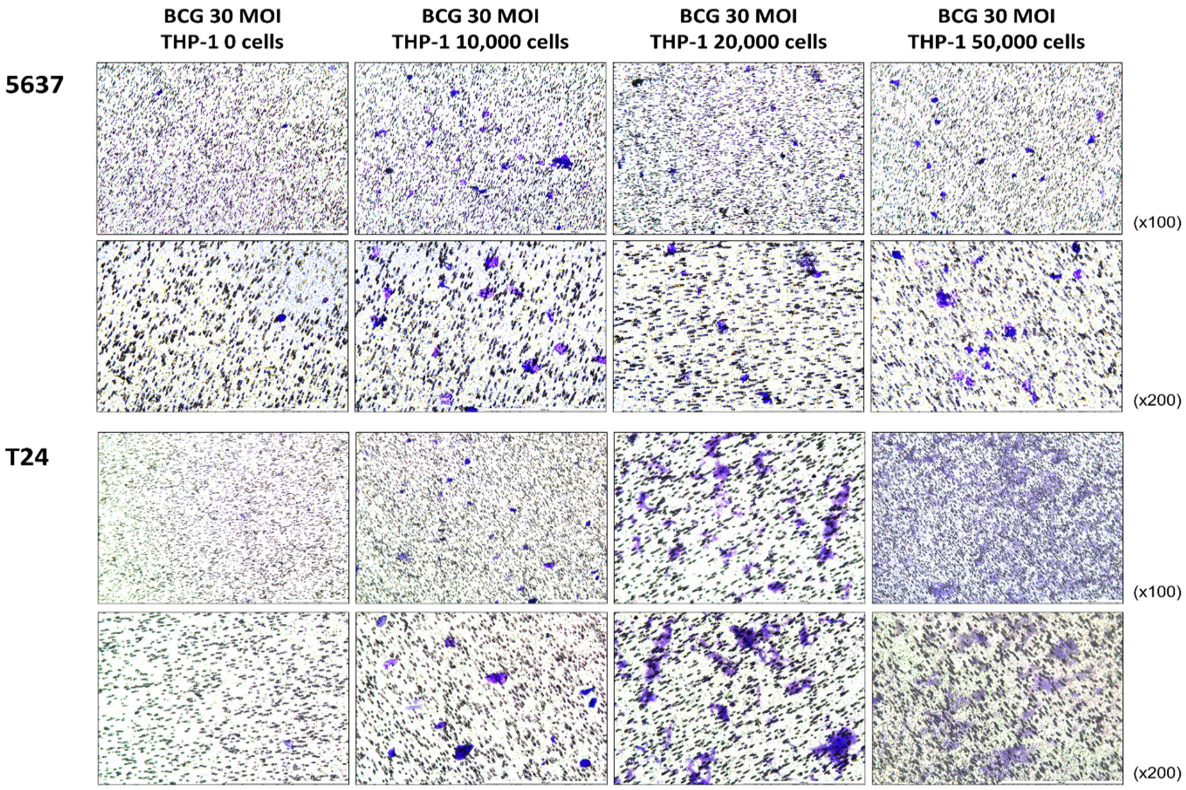

Figure S7. GelMA structure and viability of the bladder cancer cells in 3D mono- and co-culture.

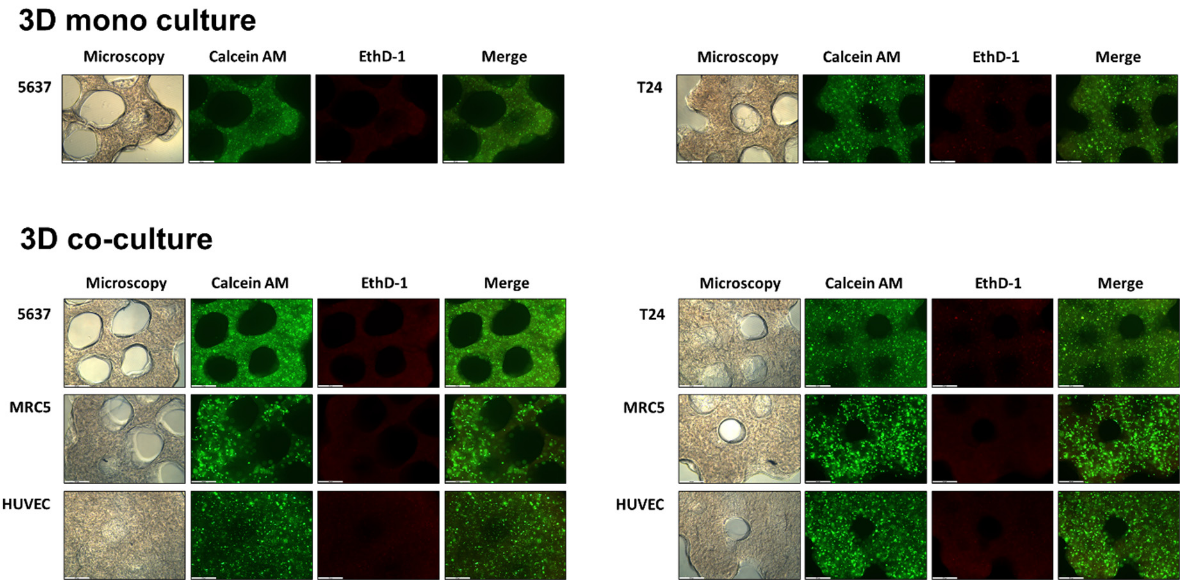

Table S1. Concentrations of growth factors in 3D mono- and co-culture. Data are presented as the mean ± SE of the mean (n = 3 per group).

5637	Mono culture				Co-culture			
	0 hours	3 hours	6 hours	24 hours	0 hours	3 hours	6 hours	24 hours
GM-CSF (pg/mL)	12.5±0.4	13.0±0.0	13.0±0.0	13.0±0.0	12.5±0.4	13.3±0.5	13.0±0.0	13.0±0.0
PDGF (pg/mL)	17.5±0.4	19.5±0.2	19.0±0.0	19.0±0.2	17.5±0.4	19.8±0.5	18.3±0.1	18.8±0.4
VEGF (pg/mL)	3095.5±17.3	2368.2±132.3	2577.7±107.2	2946.6±47.9	3095.5±17.32	2500.8±109.1	2226.8±25.8	2657.5±98.4
TGF-ß (pg/mL)	12.5±0.4	66.9±1.5	65.8±1.0	64.8±1.2	12.5±0.4	65.7±2.6	60.3±2.2	62.3±2.8

T24	Mono culture				Co-culture			
	0 hours	3 hours	6 hours	24 hours	0 hours	3 hours	6 hours	24 hours
GM-CSF (pg/mL)	12.5±0.4	13.0±0.0	12.4±0.2	12.2±0.1	12.5±0.4	13.2±0.4	12.7±0.3	12.3±0.2
PDGF (pg/mL)	17.5±0.4	18.5±0.2	18.0±0.2	17.9±0.3	17.5±0.4	18.3±0.3	17.3±0.3	17.7±0.1
VEGF (pg/mL)	3095.5±17.3	2428.3±48.4	2280.4±225.8	2526.1±63.6	3120.0±0.0	2098.7±73.0	2201.0±81.4	2210.4±53.5
TGF-ß (pg/mL)	12.5±0.4	62.5±1.6	66.2±0.3	63.3±2.1	12.5±0.4	62.8±3.0	59.7±0.4	60.8±2.5

Figure S8. Bladder cancer cell viability measured by live/dead staining at 3 days after BCG treatment at BCOC

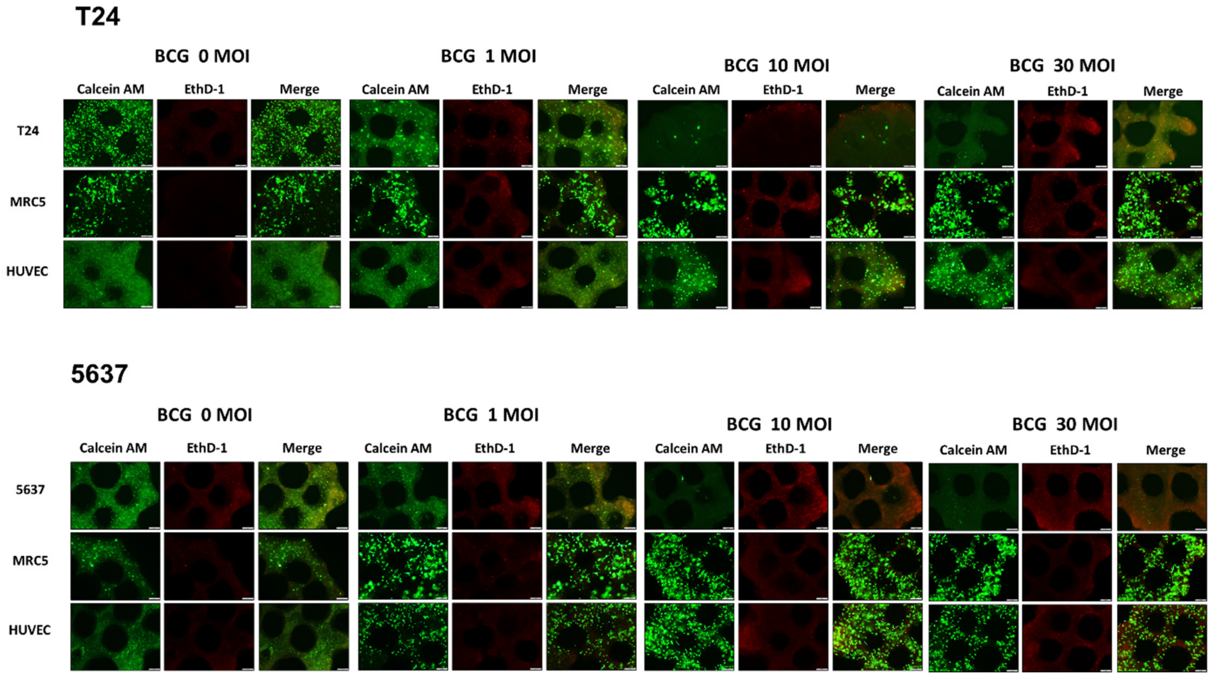

Figure S9. Migration rate of monocytic THP-1 cells in permeable membrane 24 h after BCG treatment at BCOC. Data are presented as the mean ± SE of the mean (n = 3 per group). *$p < 0.05$, **$p < 0.01$. SE: standard error.

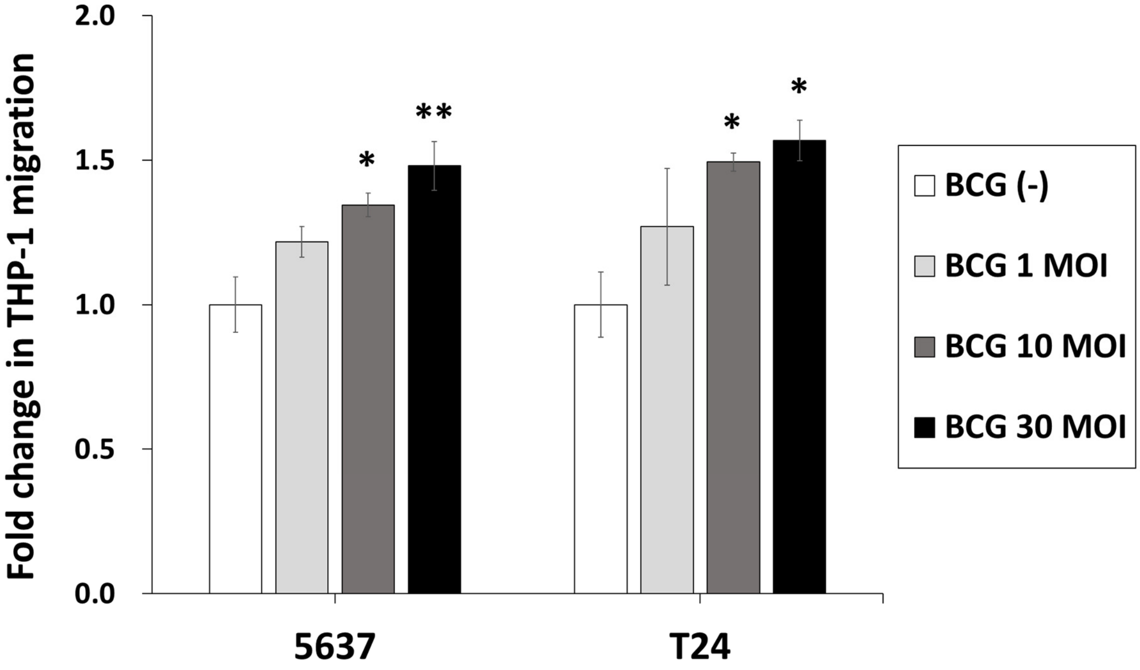

Figure S10. Comparison images of EBM-2 and other culture media for each cell lines. A, Comparison in 2D culture model; B, Comparison in 3D structural model. Migration rate of monocytic THP-1 cells was measured in permeable membrane at 24 h after BCG treatment. Scale bar shows 500µm.

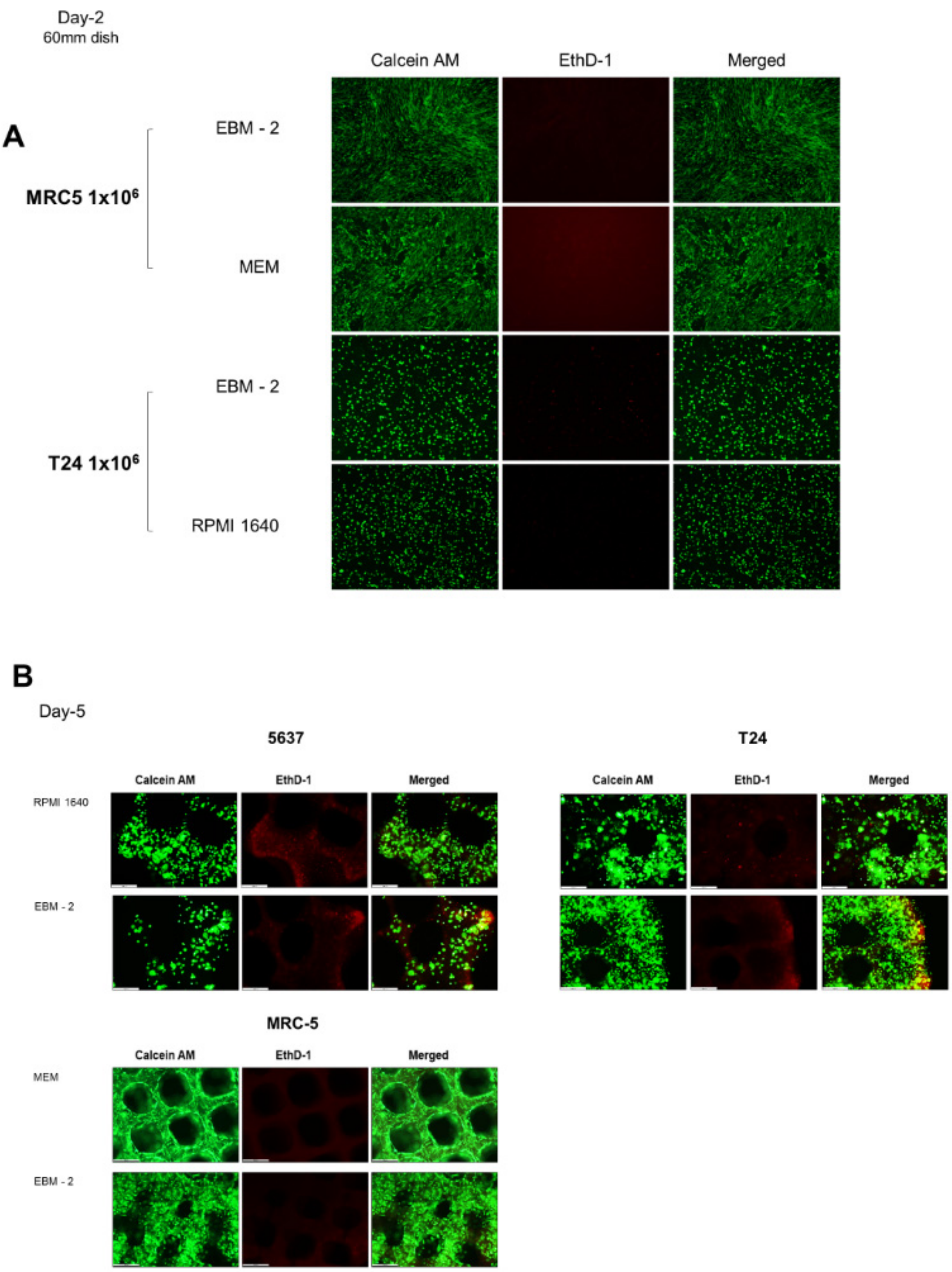

Figure S11. Microfluidic channel of BCOC. This microfluidic channel contains a cylindrical chamber (5 mm height, 6 mm diameter) for the bio-printed cell block.

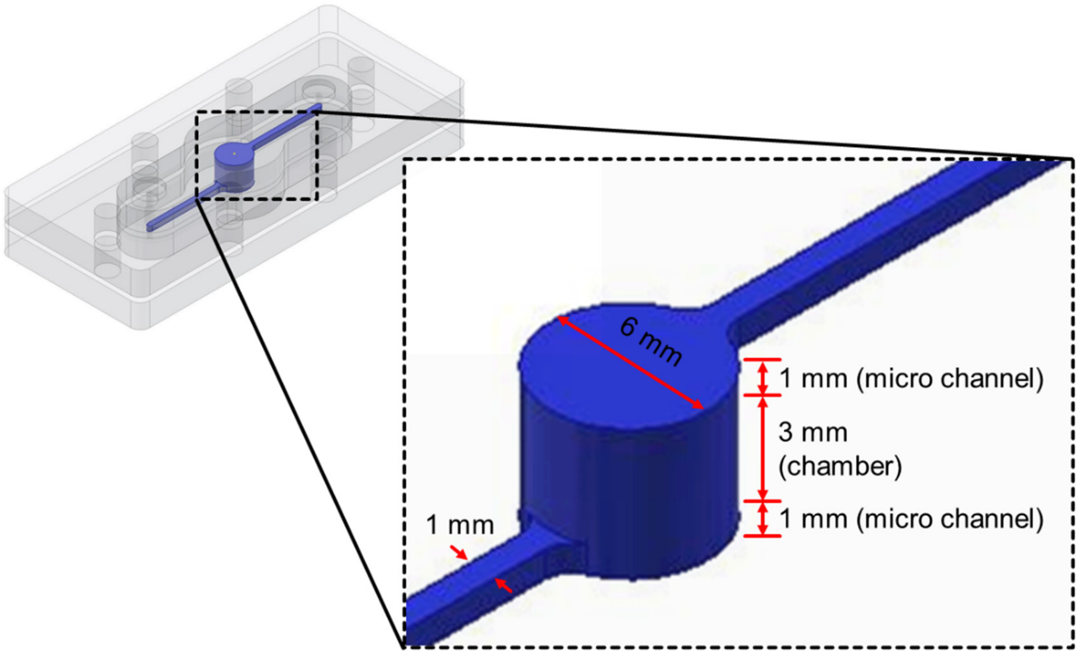

Figure S12. Computational domain of the BCOC microfluidic channel. A. Circular (upper) and square (lower) shapes of chamber were modeled. The height (5 mm) of the chamber was the same for both geometries. B. The hexahedral mesh was used. The porous medium model was applied to the bio printed cell block.

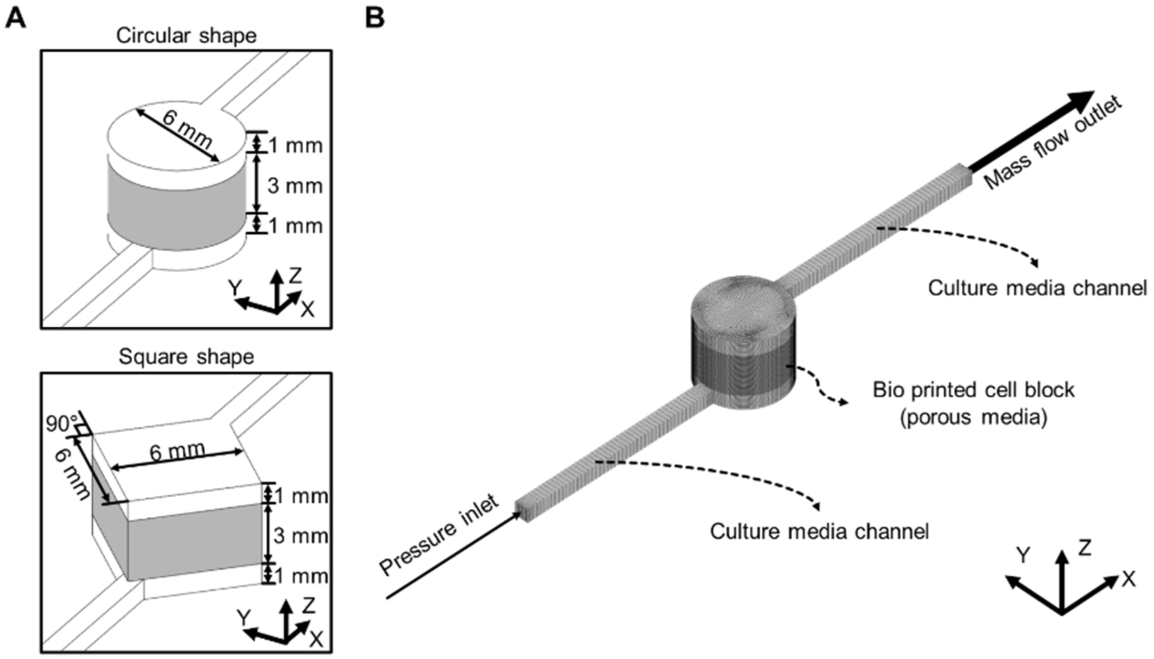

Supplement: Supplementary file 1 [file ijms-22-08887-s001.zip › ijms-1289276-supplementary.pdf]
